# Chromosome Segregation in Bacillus subtilis Follows an Overall Pattern of Linear Movement and Is Highly Robust against Cell Cycle Perturbations

**DOI:** 10.1128/mSphere.00255-20

**Published:** 2020-06-17

**Authors:** Nina El Najjar, David Geisel, Felix Schmidt, Simon Dersch, Benjamin Mayer, Raimo Hartmann, Bruno Eckhardt, Peter Lenz, Peter L. Graumann

**Affiliations:** a SYNMIKRO, LOEWE Center for Synthetic Microbiology, Philipps Universität Marburg, Marburg, Germany; b Department of Chemistry, Philipps Universität Marburg, Marburg, Germany; c Department of Physics, Philipps Universität Marburg, Marburg, Germany; d Max Planck Institute for Terrestrial Microbiology, Marburg, Germany; University of Wyoming

**Keywords:** *Bacillus subtilis*, DNA replication, chromosome segregation, computer modeling

## Abstract

Although several proteins have been identified that facilitate chromosome segregation in bacteria, no clear analogue of the mitotic machinery in eukaryotic cells has been identified. In order to investigate if recognizable patterns of segregation exist during the cell cycle, we tracked the segregation of duplicated origin regions in Bacillus subtilis for 60 min in the fastest practically achievable resolution, achieving 10-s intervals. We found that while separation occurred in random patterns, often including backwards movement, overall, segregation of loci near the origins of replication was linear for the entire cell cycle. Thus, the process of partitioning can be best described as directed motion. Simulations with entropy-driven separation of polymers synthesized by two polymerases show sudden bursts of movement and segregation patterns compatible with the observed *in vivo* patterns, showing that for *Bacillus*, segregation patterns can be modeled based on entropic forces. To test if obstacles for replication forks lead to an alteration of the partitioning pattern, we challenged cells with chemicals inducing DNA damage or blocking of topoisomerase activity. Both treatments led to a moderate slowing down of separation, but linear segregation was retained, showing that chromosome segregation is highly robust against cell cycle perturbation.

**IMPORTANCE** We have followed the segregation of origin regions on the Bacillus subtilis chromosome in the fastest practically achievable temporal manner, for a large fraction of the cell cycle. We show that segregation occurred in highly variable patterns but overall in an almost linear manner throughout the cell cycle. Segregation was slowed down, but not arrested, by treatment of cells that led to transient blocks in DNA replication, showing that segregation is highly robust against cell cycle perturbation. Computer simulations based on entropy-driven separation of newly synthesized DNA polymers can recapitulate sudden bursts of movement and segregation patterns compatible with the observed *in vivo* patterns, indicating that for *Bacillus*, segregation patterns may include entropic forces helping to separate chromosomes during the cell cycle.

## Abstract (precis)

We have followed the segregation of origin regions on the Bacillus subtilis chromosome in the fastest practically achievable temporal manner, for a large fraction of the cell cycle. We show that segregation occurred in highly variable patterns but overall in an almost linear manner throughout the cell cycle. Segregation was slowed down, but not arrested, by treatment of cells that led to transient blocks in DNA replication, showing that segregation is highly robust against cell cycle perturbation. Computer simulations based on entropy-driven separation of newly synthesized DNA polymers can recapitulate sudden bursts of movement and segregation patterns compatible with the observed *in vivo* patterns, indicating that for *Bacillus*, segregation patterns may include entropic forces helping to separate chromosomes during the cell cycle.

## INTRODUCTION

The mechanisms mediating chromosome segregation in bacteria are poorly understood. Among the first models was the origin attachment model proposed by Jacob et al. in 1963 (1), in which it was postulated that newly replicated origins are tethered to the cell envelope close to midcell and separated by growth of the cell between the attachment points. It is now clear that cell elongation in rod-shaped bacteria is not restricted to zonal growth but occurs over the entire length of the cell (2). Furthermore, movement of origins away from midcell is much faster than the rate of cell growth, discrediting the model: in Bacillus subtilis, the average movement of chromosome is 0.17 μm/min, while the length increases at a rate of 0.011 to 0.025 μm/min (3, 4). In spite of apparent evidence for dynamic movement of chromosome regions, mitosis-like mechanisms acting in bacterial cells have only been demonstrated for plasmid segregation (5) and for chromosome segregation for two species: proteins essential for the latter process are ParA and ParB in Caulobacter crescentus and ParAI and ParBI in Vibrio cholerae. The ParAB system appears to “pull” the duplicated origin region to the opposite cell pole, where it is anchored similarly to its sibling that remains at the other cell pole (6, 7). ParB is a CTPase that binds to DNA sites close to the origin regions (in some cases to several sites), whose binding is controlled by ATPase ParA (8–10). ParA is thought to establish a gradient within the cell, along which movement of ParB-bound DNA is mediated in a DNA-relay like mechanism (11, 12). The *parAB* genes (*soj* and *spo0J*) of B. subtilis are essential only during sporulation and not during vegetative growth, and no homologous system has been found in Escherichia coli. Rather, several different organizational systems have been found in E. coli and in B. subtilis that are involved in chromosome division: a group of small basic proteins termed nucleoid-associated proteins (NAPs), structural maintenance of chromosomes (SMC) complex MukBEF, and MatP, which organizes the terminus region on the chromosome, play important roles in chromosome segregation, while in B. subtilis, SMC/ScpAB (counterpart of MukBEF) and NAP HBsu confer important functions (13). Clearly, very different sets of proteins have evolved to ensure high fidelity of segregation (14), but a true motor-like apparatus has not been identified.

The extrusion-capture model for the processes of DNA replication and segregation, which occur concurrently, proposes that the energy released during replication is harnessed to power, at least in part, partitioning of newly duplicated chromosomal regions (15, 16). In this model, the replisome pulls the DNA template into the cell center, duplicates it, and then releases the products into opposite cell halves. Movement away from the cell center is orderly, guided by proteins involved in chromosome organization, compaction, and supercoiling. A transient association of newly duplicated DNA with the inner face of the membrane, as suggested for E. coli (17), might facilitate origin migration. Segregation could additionally be aided by the concerted activity of RNA polymerases (RNAP) (18), because transcription of most genes in B. subtilis (and most highly transcribed genes in E. coli) is oriented away from *oriC* regions, and the force generated during transcription by a single stationary RNA polymerase is ∼25 pN (19), more powerful than either myosin or kinesin motors. Together, DNA and RNA polymerases might contribute to template movement. The extrusion-capture model also postulates that once released outward from the replisome, the origins are captured and held on opposite sides of the cell, e.g., via a membrane-associated anchor. The bulk of partitioning can then be achieved by a combination of ongoing replication and chromosome recompaction. Finally, specialized mechanisms exist to ensure that sister terminus regions are not caught in the division septum (20).

Another theory suggests that entropy, by itself, is sufficient to ensure successful chromosome partitioning in bacteria (21). Polymers confined in a box can actively segregate, whereas disconnected, physically identical particles tend to mix. In polymer physics, intermingled long polymers have fewer conformational degrees of freedom, or less conformational entropy, than the ones that are completely separated. Thus, entropic forces can actively segregate mixed polymers from one another. When confinement is added to the chains, they behave like a loaded entropic spring that stores the free energy produced by the DNA-protein interaction (21–23). The idea of entropy as a driver of chromosome segregation and the importance of confinement for the success of this process were recently demonstrated by experimental data (24) from a study in which the process of chromosome replication was studied in E. coli cells with an increased width, as well as in several other studies using E. coli cells as a model system or using computer simulation (25–29).

In this study, we used fluorescence microscopy to quantify the dynamics of chromosome segregation for an entire cell cycle in 10-s intervals, allowing visualization of the chromosomal movement in B. subtilis with high temporal resolution, in search of possible recurring patterns of movement. We also visualized segregation of origin regions after induction of chromosomal double-strand breaks with the use of mitomycin C (MMC) and by inhibition of topoisomerase II, in order to assess the role of DNA damage on cell cycle arrest and continuation of replication. With the caveat in mind that cells grew slowly (under oxygen limitation and under microscopy illumination), we show that origin regions segregated in random patterns but with an overall linear fashion relative to cell growth. Computer simulation using entropic forces as a driving principle for segregation of newly replicated chromosome regions could recapitulate random but overall linear segregation patterns observed in live cells.

## RESULTS

### Experimental design.

**FIG 1:**
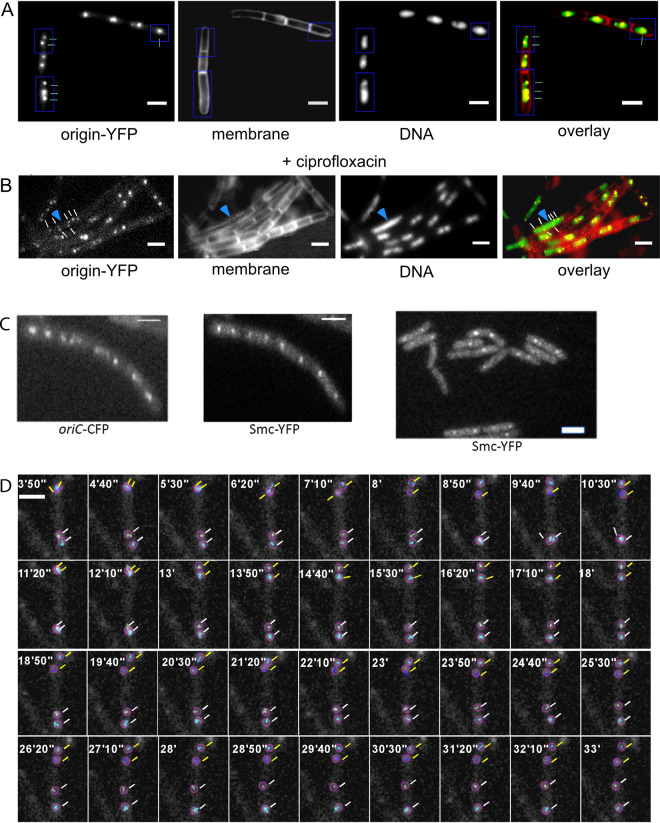
Fluorescence microscopy of FROS strains. (A) Wild-type cells expressing TetR-YFP from a constitutive promoter and carrying a *tetO* array at 353° on the chromosome (*yycR* locus). Cells with one, two, or three origin signals are indicated (note that few cells also contain 4 origin signals). Shown is a representative image from 350 cells. (B) TetR-YFP/*tetO* cells treated with 125 ng/ml of ciprofloxacin for 60 min. A cell having a replication block (elongated, several origin signals, yet a single large nucleoid) is indicated by the blue arrowhead. Shown is a representative image from 350 cells. (C) (Left two panels) Cells carrying a *lacO* array at 359° (integrated into the *spo0J* locus, which is thereby duplicated) and expressing LacI-CFP and SMC-YFP (fusion integrated at the *smc* locus as the sole source of the protein, under the control of the original promoter). (Right panel) Cells expressing SMC-YFP only (no FROS system). (D) Strain KS188 (TetR-YFP/*tetO* system) imaged with a 514-nm laser with 10-s intervals (exposure time, 200 ms); note that only invervals every 50 s are shown. The montage was done based on a movie analyzed in the TrackMate program. Tracked origins are encircled. Representative time-lapse images from 80 segregation events were captured. White bars = 2 μm.

We aimed at following the subcellular position of origin regions in very short time intervals (as short as possible) for as long as possible, covering the entire cell cycle. Fluorescent repressor/operator systems (FROS) as well as ParB/*parS* systems have been used to study various chromosome dynamics in prokaryotic and eukaryotic cells. When we employed Spo0J-yellow fluorescent protein (YFP), which binds to 10 sites surrounding origin regions in B. subtilis, we were not able to obtain time-lapse data for more than 30 min, with several exposures per second, and systems employing heterologous ParB proteins and single *parS* sites (or short tandems) yielded even lower spatiotemporal efficiency (data not shown). We therefore employed the FROS system, which has been successfully used in the past. It must be pointed out the FROS arrays have been shown to hinder the progression of replication forks to some degree and can even be used as replication road blocks when repressor proteins are highly expressed (30), such that all operator sites are saturated and fully occupied. In the two systems we have employed, repressor proteins were expressed from the constitutive “*veg*” (*lacI-cfp*) or “*pen*” (*tetR-yfp*) promoters, and large colonies were selected that grew rapidly (i.e., did not show pronounced negative effects due to the FROS system) yet allowed for chromosome sites to be efficiently visualized. In the LacI FROS system, the *lacO* cassette is inserted into the *spo0J* locus (359°) (4); in the TetR FROS system, the *tetO* array is inserted into the *yycR* locus (353°) (31). Doubling times were similar (26 ± 1 min for *lacO* at *ori*/LacI-cyan fluorescent protein [CFP]) or only somewhat lower (27.8 ± 1.5 min for *tetO* at *ori*/TetR-YFP) than that of the wild-type cells (24.8 ± 1 min) at 37°C in LB rich medium (determined from 3 independent growth experiments), or more relevant for this study, doubling time in minimal medium at 25°C was 91 ± 7 min for wild-type cells, 93 ± 7 min for the LacI-CFP/*lacO* strain, and 94 ± 5 min for cells carrying the TetR-YFP/*tetO*-FROS tag. So, for both FROS systems, there was a minimal effect on the doubling time under our experimental conditions. To further analyze if cells carrying the TetR FROS system show visible signs of cell cycle perturbation, we treated cells lacking FROS with ciprofloxacin to induce DNA replication blocks due to stalled topoisomerases. Thirty minutes after addition of ciprofloxacin, 13% of the cells contained large, nonsegregated nucleoids within elongated cells, clear sign of a replication block (Fig. 1B; 300 cells analyzed). We did not find a single such cell in cells carrying a FROS system (Fig. 1A; 350 cells analyzed) or in cells devoid of any array (310 cells analyzed). Second, we imaged Smc-YFP in cells carrying the LacI-FROS system, which have a duplicated *spo0J* gene due to the insertion of the plasmid carrying the *lacO* repeats (4), and in cells devoid of FROS and found no visible difference in the formation of Smc-YFP foci due to FROS (Fig. 1C). A total of 6.2% of cells lacking FROS did not contain any SMC-YFP foci, 8.8% contained one, 62.5% contained two, 10% three, and 12.5% four foci (*n* = 160 cells analyzed). For (*n* = 120) cells having a FROS system and expressing SMC-YFP, 7.5% showed no signal and 7.5%, 65%, 8.3%, and 11.7% had one to four foci, respectively. Because Spo0J plays an essential role in sporulation, we tested for a possible defect in the function of Spo0J, which would also affect the function of SMC and indirectly of chromosome segregation (32, 33). We found this strain to be sporulation proficient (81% ± 8% spore formation; two duplicate experiments performed) compared with cells lacking the FROS system (76% ± 5% sporulation efficiency), as was first described in reference 34. With the caveat in mind that based on the minor growth difference observed, a small number of cells may show an aberrant segregation pattern due to the FROS system, we set out to obtain information on chromosome dynamics in cells growing under microscopy conditions.

### Origin segregation follows a pattern of directed linear motion.

We tracked origin separation at 10-s intervals for 60 min or more. Figure 1 shows a typical experiment, obtained using 514-nm laser illumination and the *tetO*/TetR-YFP system, which had the best signal-to-noise ratio. In most cells growing in minimal medium, origin dynamics were characterized by one or two foci remaining localized to midcell for a highly variable period, until the replicated sister loci underwent translocation toward the quarter-cell positions (Fig. 1D). After the translocation period, *oriC* dynamics became slower and the regions usually remained at the new positions, without considerable movement. As a second caveat in our experiments, it has to be kept in mind that cells grew considerably more slowly under the microscope (180-min doubling time [*t*_D_]) than in liquid shaking culture (t_D_ = 90 min), likely because of oxygen limitation. However, cells grew exponentially after an initial lag time following mounting on the agarose pads (see Movie S1 and Fig. S1 in the supplemental material), showing that they had adapted to the less favorable growth conditions to find a new steady-state growth rate. Based on this slow growth, 19.7% of the cells (*n* = 1,120) had an origin separation event during an average experiment length of 60 min under the employed microscopy conditions. For a mixed (nonsynchronized) culture growing exponentially with a doubling time of 180 min, about 30% origin segregation events would have been expected within a 60-min imaging time. Figure S1 shows that usually after 50 min of incubation on the agarose slide, cells went from an adaptation phase into exponential growth. Thus, the lower-than-expected number of segregation events is likely due to lag time; however, a majority of cells grew exponentially under the microscope and thus segregated their origin regions in a regular manner.

10.1128/mSphere.00255-20.1 MOVIE S1 Time-lapse microscopy of B. subtilis cells growing under fluorescence microscopy conditions. Bright-field images were captured every 5 min; shown are 6 frames/s. Download Movie S1, AVI file, 8.1 MB. Copyright © 2020 El Najjar et al. 2020 El Najjar et al. This content is distributed under the terms of the Creative Commons Attribution 4.0 International license.

10.1128/mSphere.00255-20.3 FIG S1 Growth curve estimation of B. subtilis cells growing under fluorescence microscopy-based growth conditions, using bright-field imaging. The graph shows a nonparametric locally estimated scatterplot smoothing (LOESS)-based regression model, showing exponential growth for 6 representative B. subtilis cells (see Movie S1 for a field of cells). Growth is indicated by an increase of the normalized percentage of cellular area over time. The gray confidence band indicates precision defined by a range of local uncertainty in which the true regression line locally fits with 95% confidence. Estimation was done by a custom Fiji/ImageJ2 pipeline. Binary images were generated using time-lapse recordings (5-min intervals, 22 cycles) of B. subtilis cells followed by thresholding. Resulting binary information was summarized and visualized using R statistics and R Studio, respectively. Download FIG S1, TIF file, 0.4 MB. Copyright © 2020 El Najjar et al. 2020 El Najjar et al. This content is distributed under the terms of the Creative Commons Attribution 4.0 International license.

**FIG 2:**
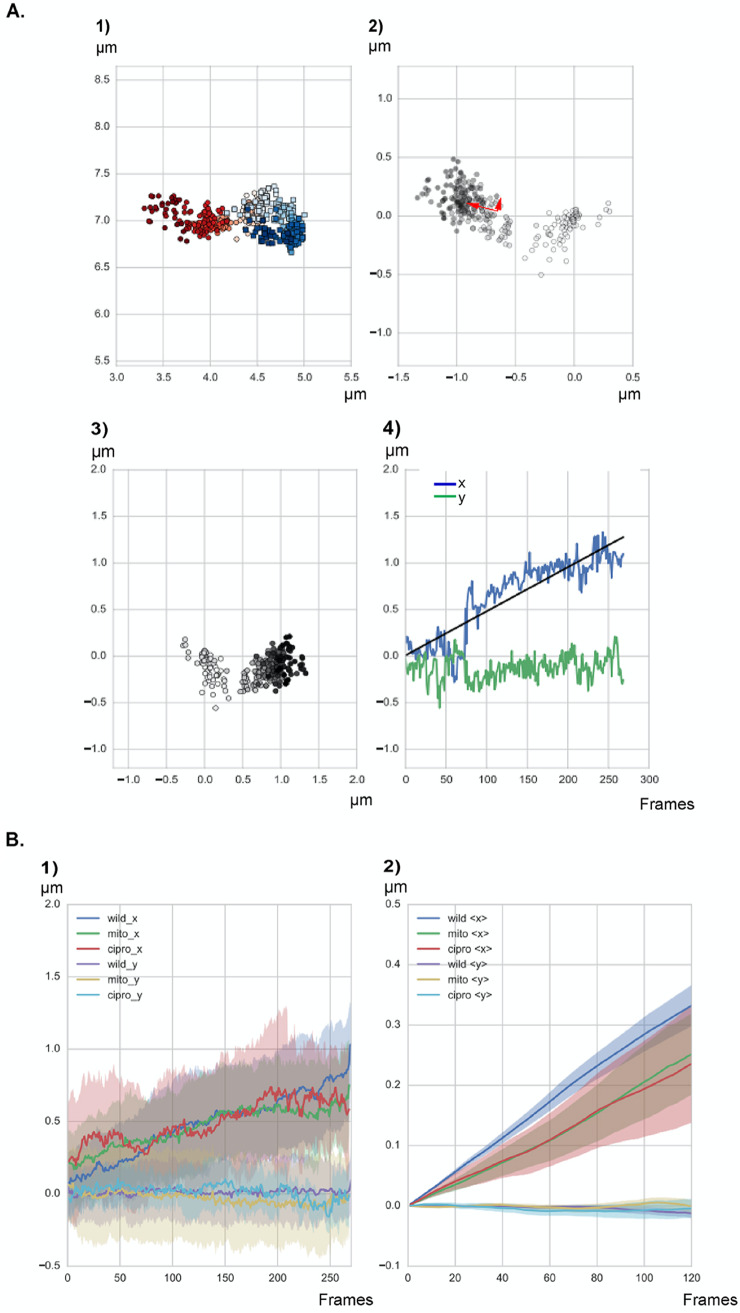
Graphs describing the separation of two origins in a representative cell. (1) Position in the cell of all the points in the tracks. The first origin is shown in red, the second in blue. Colors go gradually from bright to dark along time. (2) Increasing distance between the two origins; darker dots reflect the distances in later time points, the same shading convention as in subpanel 1. The red arrows indicate a set of orthogonal vectors pointing along the short and long axis of the cloud of points. (3) Projection of the distances in subpanel 2 on a horizontal cell 2 μm in length, coordinate system with *x* parallel to the long, along the length of the cell, and *y* perpendicular to *x*, along the width of the cell. (4) Distance between the origins along the *x* and *y* axes versus time (1 frame = 10 s). The black straight line is a trend line for linear separation in time along the *x* direction. (B) Movement over time for all tracks generated under all three different conditions. Shown are results for exponentially growing cells (wild; *n* = 80 segregation events, from five independent biological replicates), cells treated with 50 ng/ml of mitomycin C (mito; *n* = 65 segregation events from three biological replicates), and cells treated with 125 ng/ml of ciprofloxacin (cipro; *n* = 62 segregation events from three biological replicates). “*x*” and “*y*” indicate movement along the long axis (“*x*”) or the width (“*y*”) of the cells. (1) Distance between the origins along the x and *y* axes versus time. (2) Distance moved in all tracks along the *x* and *y* axes at every time lag Ʈ shown in frames. In panel B, solid lines reflect averages of all graphs for every condition.

**FIG 3:**
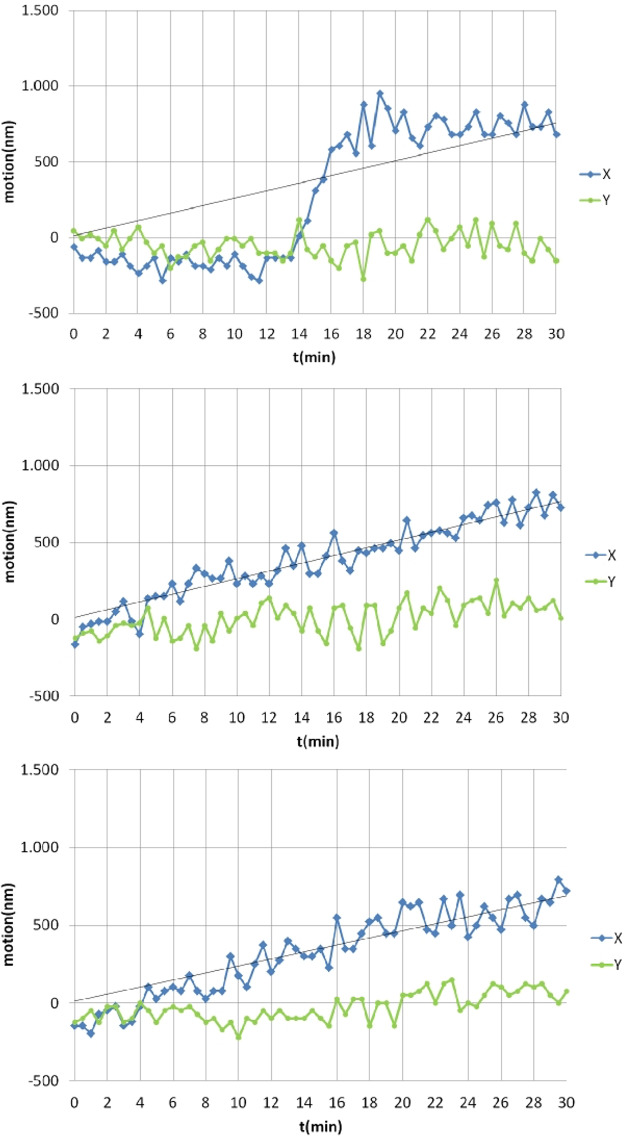
Analysis of three representative origin segregation events of cells expressing Spo0J-YFP from the original gene locus, binding to 10 sites surrounding origin regions on the chromosome.

**FIG 4:**
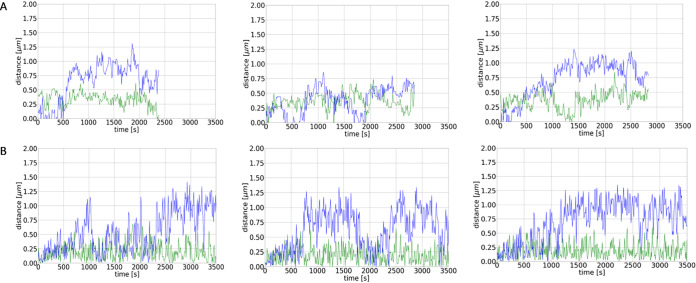
(A) Example tracks of the distance between *ori*s as a function of time as measured in the experiments. Blue lines indicate distance in *x* direction (length of cell), green in *y* direction. (B) Example tracks of the distance between *ori*s as a function of time as obtained from the numerical simulations. Shown are the distances between the *ori*s of the old and new DNA as a function of time for different runs of the same simulation with the chromosome being composed of 80 beads, each with a radius of 0.075 μm.

For the quantitative analysis of the movement, all the separation events of origins in an imaging field were tracked, from at least three independent replicates. This resulted in 221 separation events, of which 80 were completely uninterrupted by loss of the focal plane or bleaching events. The 80 events are thus a good representation of usual segregation patterns. An example of the data that were extracted is shown in Fig. 2. Figure 2A-1 shows positions of the origins, one in blue and one in red. Figure 2A-2 shows the distance between the two origins, obtained as a difference in position vectors; early times are light gray and later times are in darker shades. This collection of points was then transformed to a coordinate system with one axis along the longest extension and one perpendicular to it along the shorter extension (indicated by the red arrows in Fig. 2A-2). This way the movement of the microscope stage or of cells on the agarose surface did not affect the relative movement of origins. The transformed data are shown in Fig. 2A-3, and time traces of the positions along the long (*x*) axis (blue) and the shorter transverse (*y*) axis (green) are shown in Fig. 2A-4. It is clearly visible that there was considerable separation along the *x* axis (cells were between 2 and 4 μm long) but not along the *y* axis (1-μm width).

The acquired movies show that separation of origins occurs in a seemingly stochastic manner; a consensus pattern of movement was not detectable. Some segregation events were subjectively similar. For example, in 6% of cases origins separated but then moved back toward each other; in 15%, origins separated quickly and remained separated; and in 5%, an apparent jump in separation was followed by a gradual decline toward the end. In many cases, origin separated rather gradually (25%); in the remaining 49% of events, various variations of segregation jumps and intervals of back movement were apparent (Fig. S2). In these 49% of cases, we also observed events in which separation and backward movement occurred repeatedly for some time until segregation finally became permanent. The 80 segregation events we monitored did not resemble each other in a striking manner, so we were not able to observe any particular pattern. It has to be kept in mind that cells had gone through a transition to adapt to low-oxygen conditions and that some cells will encounter replication arrest, which may explain some of the observed heterogeneity of segregation patterns. However, since the patterns were observed in cells that continued to grow exponentially under imaging conditions, it is interesting that segregation occurred in an astonishingly stochastic manner.

10.1128/mSphere.00255-20.4 FIG S2 Analysis of several origin segregation events in cells carrying a TetR-YFP/*tetO* system to label origin regions on the chromosome. Download FIG S2, TIF file, 1.6 MB. Copyright © 2020 El Najjar et al. 2020 El Najjar et al. This content is distributed under the terms of the Creative Commons Attribution 4.0 International license.

In earlier work using 5-min acquisition intervals, it was noticed that many segregation events occur faster at the beginning and show a plateau toward the end (4). Using much faster acquisition and many more time intervals, we were able to show that segregation events do not contain any characteristic patterns, except for an overall efficient separation of duplicated regions throughout the length of acquisition periods.

Figure 2B-1 summarizes information about the tracks of all analyzed separation events. It can be seen that in the mean, separation between the origins grew gradually in time, with an essentially linear time dependence. Importantly, plotting the displacement in micrometers against a lag time Ʈ for all tracks (where Ʈ = *n*Δ*t*, with Δ*t* being the time delay between the consecutive frames and *n* being the interval of frames over which the distance is measured and averaged) shows a clear, directed movement of origin regions (Fig. 2B-2). Movement along the *y* axis is highly restricted, underlining the directionality in movement along the length of the cells. Therefore, chromosome segregation clearly follows an overall pattern of directed motion, with no identification of a recurring motif of a defined segregation event.

As a control, we performed similar experiments using a strain expressing Spo0J-YFP from the original gene locus to follow the movement of origin regions. Figure 3 shows that we obtained segregation patterns that differed from each other, including seemingly random segregation patterns that were also observed using the TetR/*tetO* FROS system (Fig. 4 and Fig. S2). Based on this alternative *in vivo* labeling method (*n* = 20 segregation events, with 22% of cells showing segregation events during 60-min experiments), data acquired using the FROS system likely represent events that are reflective of unperturbed segregation.

### MD simulation based on entropy-driven separation of newly synthesized polymers can recapitulate observed segregation patterns.

**FIG 5:**
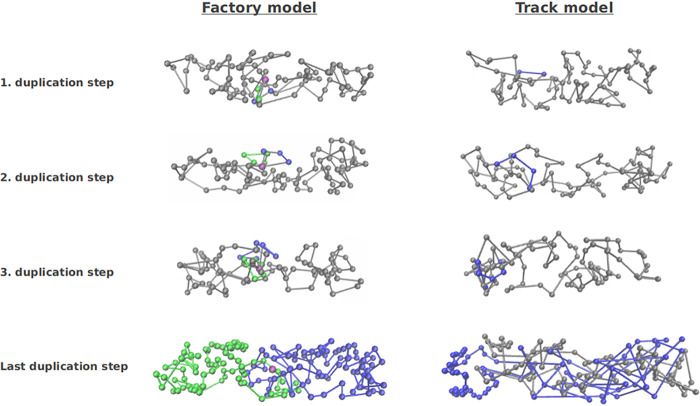
Different stages of the replication process as obtained from the numerical simulations. The duplication steps mark the events at which two new blobs are built by the two polymerases. Results shown are for the factory model (left) and for the track model (right). In the latter, replication is not confined to a specific cellular region as the replication polymerases move along the chromosome. In the factory model, the polymerase is assumed to have a fixed cellular position, which is marked by the purple sphere (16, 67).

**FIG 6:**
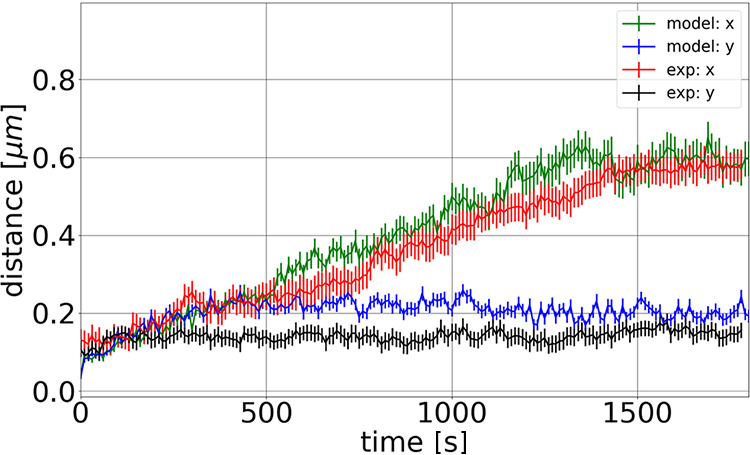
Averaged distances of the *ori*s of the old and new chromosomes during the replication period. Results shown are for averages over 80 experimental trajectories (red line) and over 80 simulation trajectories (green line). The standard errors of the means are shown as vertical error bars. In both, the experiments and the simulations the *ori*s segregate within the long axis of the cell (*x* axis), while their distance along the *y* axis does not change notably. It can be seen that the trajectories overlap in large parts within the standard errors and that the average distance of the *ori*s at the end of replication is in both cases between 0.5 and 0.6 μm.

In our simulations, we represented the bacterial chromosome as a spring connected chain of spherical beads, each containing compacted DNA. The starting configuration of the simulations is a circular chromosome which is then replicated. As mentioned above, for replication we implemented two different models, the track model and the factory model (see Fig. 5, Text S1, and Fig. S3, S4, and S5). The comparison of simulations with the biological experiments revealed a better agreement of the latter with the factory model. The factory model as well as the experimental data revealed overall linear segregation by averaging over many tracks. In contrast, for the track model the averaged segregation was not linear, but origins experienced an oscillatory segregation with periods in which they moved toward each other and periods in which they moved apart from one another, which were still visible in the averaged segregation graph. Therefore, the factory model was used for further analyses. In both models, replication is divided into several duplication steps in which always two beads are duplicated by the replication polymerases. Between these duplication events the chromosomes exhibit thermal fluctuations giving rise to an effective entropic repulsion between the full chromosome and the partially replicated chromosome. As a consequence, the chromosomes start to separate during replication. While intermediate configurations are still mixed (see Fig. 5), the two chromosomes are almost fully separated by the time replication is completed. Figure 4B shows different simulations of origin separation based on 80 beads using the factory model. The simulations show that the origins almost completely move to the opposite poles in the cell during duplication due to the thermal fluctuations and resulting entropic repulsion between the beads. Nevertheless, there are also several trajectories in which the origins do not separate during the replication period. This is also found in the experimentally monitored trajectories; see Fig. 4A (for more examples, see Fig. S6). Thus, we note a high variability between the single trajectories in both simulations and experiments. Importantly, the different bursts of segregation events, returns to smaller distances, and different overall velocities of separation closely matched experimentally observed segregation patterns (Fig. 4A), revealing that entropy-driven simulations provide a good model for actual events. For further comparison of the simulations with the experimental trajectories, Fig. 6 shows the average trajectories for the experimental results and the simulations (average over 80 trajectories in both cases). The standard errors of the means are shown as vertical error bars. As can be seen in Fig. 6, the averages are in good agreement, indicated by the overlap of the curves. Furthermore, the final distance between the origin regions is in the range of 0.5 to 0.6 μm in both cases. For further analyses, more experimental trajectories would be needed to allow a direct comparison of the different classes of trajectories (e.g., fast trajectories, slow trajectories, etc.).

10.1128/mSphere.00255-20.2 TEXT S1 Calculation of time scale within MD simulations, entropic equilibration of confined polymers, implementation of different replication models, variation of replication intervals, variation of ϵ within simulations, and spatial organization of origins. Download Text S1, DOCX file, 0.03 MB. Copyright © 2020 El Najjar et al. 2020 El Najjar et al. This content is distributed under the terms of the Creative Commons Attribution 4.0 International license.

10.1128/mSphere.00255-20.5 FIG S3 Before replication and segregation of the chromosome are started in the simulations, the initial chromosome configuration is equilibrated by integrating the system for 10^3^ time steps (10^3^ × 0.01τ = 100τ). This is done to prevent initial high repulsion forces caused by too-close distances between adjacent beads. The graph shows an example of the monitored energies during this equilibration procedure. Shown are the kinetic energies of the beads (blue line), the energies due to the bonded interactions of the springs connecting the beads (green line), and the nonbonded interaction energies (red line) resulting from the interaction with the WCA potential. The sum of these terms gives the total energy (black line). As can be seen, the energy fluctuates around a constant value after the equilibration procedure. Download FIG S3, TIF file, 0.4 MB. Copyright © 2020 El Najjar et al. 2020 El Najjar et al. This content is distributed under the terms of the Creative Commons Attribution 4.0 International license.

10.1128/mSphere.00255-20.6 FIG S4 Example run showing the segregation of two initially overlapping chromosomes, each consisting of 80 beads. (Left) Degree of separation as function of simulation time τ. The capital letters mark the positions at which the snapshots are taken (right). As can be seen, the chromosomes need a relatively long time to start segregation, which typically sets in when the chromosomes form overhanging regions filled by beads of different chromosomes (snapshot C). From this point on, segregation proceeds rather fast until the chromosomes are fully segregated (snapshot E). Download FIG S4, TIF file, 0.9 MB. Copyright © 2020 El Najjar et al. 2020 El Najjar et al. This content is distributed under the terms of the Creative Commons Attribution 4.0 International license.

10.1128/mSphere.00255-20.7 FIG S5 Averaged segregation times for two initially overlapping chromosomes as shown in Fig. S4. Shown are the times needed for entropic separation of chromosomes consisting of 20, 40, 50, 60, and 80 beads. Each data point is the result of an average of 10 simulation runs. The red line shows a polynomial fit to these results. With this fit we interpolated the theoretical value of the time needed for entropic separation of two beads. Download FIG S5, TIF file, 0.1 MB. Copyright © 2020 El Najjar et al. 2020 El Najjar et al. This content is distributed under the terms of the Creative Commons Attribution 4.0 International license.

10.1128/mSphere.00255-20.8 FIG S6 (A) Example tracks of the distance of *ori*s as a function of time as measured in the experiments. Blue lines indicate distance in *x* direction (length of cell), green in *y* direction. (B) Example tracks of the distance of *ori*s as a function of time as obtained from the numerical simulations. Shown are the distances between the *ori*s of the old and new DNA as a function of time for different runs of the same simulation with the chromosome being composed of 80 beads, each with a radius of 0.075 μm. Download FIG S6, TIF file, 1.1 MB. Copyright © 2020 El Najjar et al. 2020 El Najjar et al. This content is distributed under the terms of the Creative Commons Attribution 4.0 International license.

**FIG 7:**
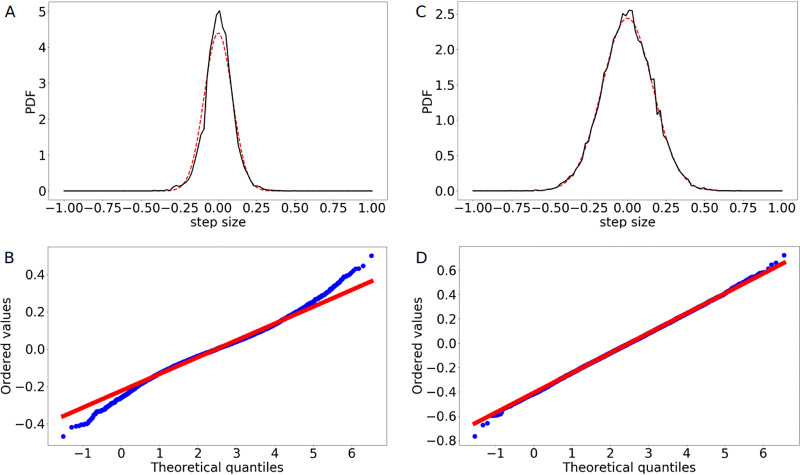
Analysis of the step size distribution of the *ori* movement. (A) Calculated PDF of the step size distribution along the longitudinal axis of the cell for the experimental data (black line) compared to a fitted normal distribution (red line). (B) Probability plot comparing the experimental data (blue points) to a normal distribution (red line). (C) Calculated PDF of the step size distribution along the longitudinal axis of the cell for the simulation data (black line) compared to a fitted normal distribution (red line). (D) Probability plot comparing the simulation data (blue points) to a normal distribution (red line). While the numerical results can be well represented by a Gaussian distribution, the experimental one differs from a normal distribution, as can be seen at the tails of the probability plot. However, the plot shows a reasonably linear pattern in the center of the data. The deviations could be due to experimental difficulties in tracking slow movement. Step size distributions were calculated from 80 individual runs.

Finally, for further comparison between simulations and experiments, we have analyzed the step size distributions of the trajectories (Fig. 7). We plotted the calculated probability density functions (PDFs) of the data and applied a Gaussian fit function. To test the goodness of the fit, we also used probability plots, where the corresponding cumulative distribution functions (CDFs) are plotted against each other. As can be seen from Fig. 7C and D, in the simulations the step size distribution is well represented by a normal distribution, as expected for a process of entropic segregation (35). However, the experimental step size distribution (shown in Fig. 7A) differs from a Gaussian distribution (Fig. 7B). While the probability plot shows a reasonably linear pattern in the center of the data, we found departures from the fitted red line of a normal distribution at the tails of the experimental data. In particular, the high number of small steps caused the deviations here. This could be due to experimental difficulties in the tracking procedure and the limit of resolution of light microscopy (250 nm in this case). Note that we analyzed the effect of changing the value of ϵ within our simulations (Fig. S7) and analyzed the results of the simulations for the final positions of the two origins after replication (Fig. S8), detailed in Text S1.

10.1128/mSphere.00255-20.9FIG S7Averaged velocity of the faster-moving *ori* along the longitudinal axis of the cell as a function of different values for ϵ within the simulations. For each parameter setting, 80 runs were performed and their results were averaged. Download FIG S7, TIF file, 0.2 MB.Copyright © 2020 El Najjar et al.2020El Najjar et al.This content is distributed under the terms of the Creative Commons Attribution 4.0 International license.

10.1128/mSphere.00255-20.10 FIG S8 Final positions of the two *ori*s after replication (3,300 s) shown in a normalized cell as predicted by the model. The averaged final position of *ori*1 is 0.61, and the averaged final position of *ori*2 is 0.38. Download FIG S8, TIF file, 0.3 MB. Copyright © 2020 El Najjar et al. 2020 El Najjar et al. This content is distributed under the terms of the Creative Commons Attribution 4.0 International license.

### Origin movement is robust against cell cycle perturbation.

**FIG 8:**
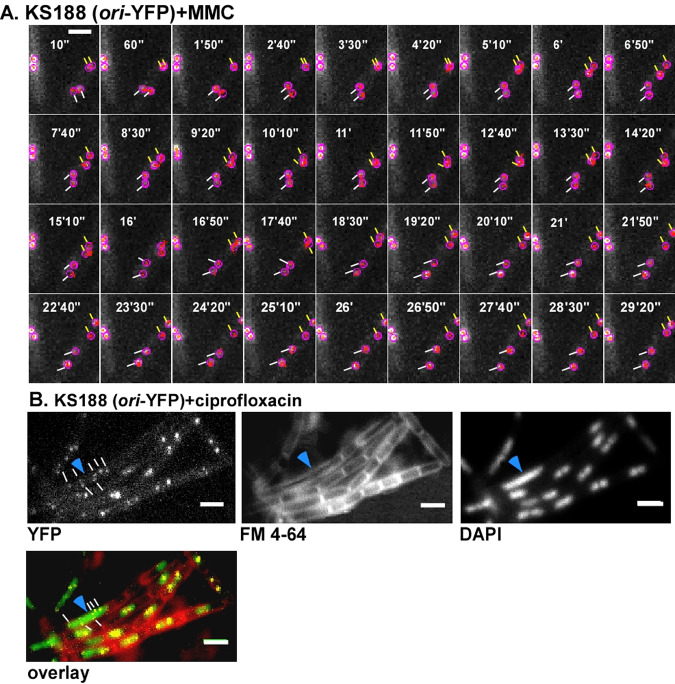
KS188 cells (TetR-YFP FROS system) imaged with the 516-nm laser with 10-s interval under two different types of stress: 50 ng/ml of MMC for 15 min in panel A (example of 65 segregation events done in three independent biological replicates) and 125 ng/ml of ciprofloxacin for 15 min in panel B. The montage in panel A was done based on a movie analyzed in the TrackMate program. Tracked origins are encircled, and local tracks, not entire tracks, are shown within the circles in order to avoid crowding in the image. Lines of the same color show a couple of separating origins. Panel B shows an example of cells (total of *n* = 310 from three independent biological replicates) after ciprofloxacin treatment. Most cells show several origins (white lines), and an elongated cell with an unsegregated nucleoid, characteristic of a block in DNA replication, is shown with a blue arrow. White bar = 2 μm.

Mitomycin C (MMC) leads to the formation of DNA adducts and to interstrand cross-links, which slow down replication and are usually repaired via homologous recombination. Following MMC treatment for 15 min, cells continued to grow and elongate, while cell division was inhibited (Fig. 8B), due to SOS induction via single-stranded-DNA (ssDNA)-bound RecA (36). On average, cells became 1.7-fold longer, 60 min after MMC addition, than nontreated cells. In every field of imaging, around 15% of cells showed no origins or a single *oriC* in the cell middle, which we assume were dead or dying cells, based on the fact that treatment of cells with 50 ng/ml of MMC for 60 min kills about 25% of cells (37). However, when we monitored *oriC* dynamics starting 15 to 20 min after addition of MMC (Fig. 8A), separation events were seen in 18% of 364 counted cells (3 replicates), which is comparable to the value for cells growing exponentially (Fig. 1). Figure 2B shows that the addition of MMC to exponentially growing cells resulted in the slowing of the separation process compared to that in the wild type, although segregation still occurred in a linear fashion. Though the origins eventually separated, they moved smaller distances in the same time window along the *x* axis than for nonstressed cells; again, the movement along the *y* axis was restricted.

To study the effects of a second strategy to slow down segregation, we used ciprofloxacin to block the activity of topoisomerase IV (topo IV). In E. coli, in the absence of functional topo IV leads to multiple catenated *ori*s clustered at midcell. Once functional topo IV was restored, the decatenated *ori*s segregated toward the cell poles (38). B. subtilis topo IV is also essential for the resolution of interlinked chromosomes following replication (39). Although DNA gyrase is the main target for quinolones in Gram-negative bacteria, topo IV is the preferred target in some Gram-positive organisms, such as B. subtilis, and trapping of gyrase occurs at much higher drug concentrations (40–44). In order to mainly block topo IV, we used 0.125 μg/ml of ciprofloxacin, the lower recognized MIC for B. subtilis (45, 46). Around 1 h after the addition of ciprofloxacin to the growing culture, we noticed that the cells had multiple origins, mostly 4 and occasionally 5 to 8 (an average of 5 per cell [*n* = 200], versus 2.5 under wild-type conditions). Of note, reduction of the activity of DNA gyrase, which will be inhibited to a minor degree by the employed concentration of ciprofloxacin, will lead to overinitiation of replication (47). Many of the cells had a clear segregation defect, and around 10% of the cells were either anucleate or had unsegregated nucleoids (Fig. 8B). However, the rate of origin separation during topo IV inhibition (movies were started 15 to 20 min following addition of ciprofloxacin) was at 20.6% (*n* = 218 cells), almost the wild-type level, which shows that despite the damage, the cells were still able to separate their origins (Fig. 2B). Figure 2B shows that the speed of separation was affected by the addition of ciprofloxacin but only marginally. The distance moved by the separating origins along the long cell axis was smaller than that for the wild type, but higher than for mitomycin C for the time window of 30 min. Therefore, origin separation is highly robust against perturbations and allows cells to proceed with their cell cycle, albeit somewhat slower than in nonstressed cells.

## DISCUSSION

An important goal of this work was to analyze chromosome segregation in a bacterium over an entire cell cycle with the fastest possible acquisition rate, in order to probe if the dynamics of segregation of origin regions on the chromosome reveal a conserved pattern of movement that may provide insight into the question of whether the process may be driven by a motor-like mechanism. Using laser-based epifluorescence microscopy including electron-multiplying charge-coupled-device (EM-CCD) camera technology, we were able to capture a high number of segregation events in 10-s intervals, where previously, information of these events had been available for 5-min intervals. We saw many examples for apparently stochastic segregation patterns, sometimes including big jumps, reversals in segregation, as well as strongly differing speeds of movement of origins away from each other. We failed to find any pattern that was recurring or conserved between the captured segregation events.

One explanation for the highly heterogeneous segregation patters could be our use of a bulky fluorescence tag (FROS system) and cells that have gone through an adaptation phase of oxygen limitation. However, the growth rate of the FROS-bearing cells was only 10% lower than that of wild-type cells (repressor proteins must be highly induced to convert FROS systems into replication roadblocks) in liquid culture, and cells continued to grow under microscopy conditions, suggesting that many cells grew under favorable conditions and, albeit at a low rate, continued with their cell cycle.

An alternative explanation for our results is the idea that entropy-driven diffusion could be a driving force in chromosome segregation, as was suggested by Jun and Mulder (22). This would mean that segregation involves an overall stochastic nature and directed diffusion-like behavior. Indeed, on average, our data show an essentially linear separation over long periods, while individual separation tracks showed striking variations and large fluctuations. While we cannot quantify the extent of noise that the fluorescence labeling system and growth conditions introduced into our system, we can conclude that overall, our data are supportive of entropy-driven diffusion and in disfavor of coordinated, motor-like movement.

We wished to investigate if entropy could explain observed segregation patterns and overall efficiency for segregation of origin regions, and we employed molecular dynamics simulations of a bead-spring model for DNA. Our analyses revealed different patterns of segregation events and a separation time span that was mostly achieved by the time replication was completed. The separation time span was similar to that seen in our *in vivo* experiments. The observations are thus in agreement with the assumption that segregation could be governed by stochastic events of entropy driven separation of DNA polymers.

Several factors have been identified to contribute to chromosome dynamics in bacteria, including RNA polymerase (18), which can move DNA like a motor protein as it transcribes, and ParA-like proteins (including MinD) (12, 48) that appear to act in a ratchet-like manner (49). Interestingly, structural maintenance of chromosomes (SMC/MukB) proteins (50–52) can actively extrude DNA loops *in vitro* (53, 54) and play a crucial role in segregation in many bacteria. Therefore, bacteria have evolved several mechanisms that can provide a driving force for DNA segregation. Our data argue that entropy may also play an important role as a driving force of chromosome segregation in B. subtilis and possibly in other bacteria. This idea is in full agreement with data obtained for E. coli cells, in which entropic forces have been proposed to play an important role in the organization of chromosome regions and of MukB (SMC orthologue) centers (25) and in MinD-facilitated chromosome segregation (48). Recent findings from observing replication and chromosome segregation in E. coli cells with a larger diameter also point toward movement of duplicated origins from more condensed toward less condensed regions, using entropy. Random segregation increases the more cell width increases and the more guiding of origin movement by the rod-shaped cell membrane is thereby reduced (24). The harnessing of physical polymer-based forces to drive segregation is highly efficient and elegant, and it removes the need for a complex segregation machinery, which would further put constraints on replication and transcription on the chromosome. Also, energy investments for an active machinery can be avoided. It should be noted that many bacteria lack SMC proteins or segregate their chromosomes almost normally in spite of carrying an *smc* deletion (55). Thus, SMC/MukB complexes and ParAB systems may contribute to the motion and/or directionality of segregation in bacteria to very different degrees, while entropy-driven segregation may be a fundamental basis of this process.

A second important finding of this work is the high robustness of the B. subtilis cell cycle against cell cycle perturbations with respect to chromosome segregation. Inhibition of gyrase activity or induction of DNA damage slowed down chromosome partitioning but maintained the overall linear progression of origin movement away from the midcell. Clearly, cells needed more time to segregate origin regions, due to fixing of DNA breaks and replication fork restart events, but cells never showed extended periods of segregation arrest. These findings show that the underlying mechanism of segregation can withstand replication obstacles and thus seems not to require stringent checkpoints, other than SOS response-induced division arrest, which gives cells more time to deal with slowed replication. We propose that as long as cells continue to replicate their DNA at a decent rate, entropic forces are able to provide a robust segregation mechanism. We are aware that our findings and suggestion might go against current thinking and believe that it is all the more important to keep an open discussion until one or the other models are disproven.

## MATERIALS AND METHODS

### Bacterial strains.

Bacterial strains used in this study are given in Table 1. Data shown in this work are all from at least three independent biological replicates.

**TABLE 1:** Strains used in this study

Strain	Genotype	Reference(s)
PG26	*spo0J* (359°)::*lacO* cassette (Cm^r^, 359°) *thrC*::*lacI-cfp* (Mls^r^)	15, 16
AT62	*thrC*::*lacI-gfp* (Mls^r^) *cgeD*::*lacO* cassette (Cm^r^, 181°)	3, 4
KS188	*yycR* (353°)::*tetO* array (Kan^r^) *cgeD*::*P_pen_ tetR-yfp* (Tet^r^)	68

### Fluorescence microscopy.

To quantify foci under slow growth conditions, B. subtilis cells were grown in S7_50_ minimal medium not supplemented with amino acids at 25°C under shaking conditions until exponential growth. For the quantification under fast growth, cells were grown LB and incubated in a shaking water bath at 37°C. Three microliters of cells was then transferred on an agarose slide-glass slide (microscope slides standard; Roth) coated with an agarose layer (S7_50_ minimal medium, 10 mg/ml of agarose) and covered with a coverslip (Roth). Conventional light microscopy was performed using a Zeiss Observer Z1 (Carl Zeiss) with an oil immersion objective (magnification, ×100; numerical aperture, 1.45; alpha Plan-FLUAR; Carl Zeiss) and a CCD camera (CoolSNAP EZ; Photometrics). Cells were treated with red fluorescent membrane stain FM 4-64 (final concentration, 1 nM) and DNA intercalating blue fluorescent dye 4′,6-diamidino-2-phenylindole (DAPI; final concentration, 0.72 nM) and incubated for 2 min at room temperature prior to microscopy. Filter sets used were as follows: DAPI, AT 350/50, T 400 LP, and 460/50 ET; CFP, 438/24, 458HC, and 483/32 HC; YFP, 500/20 ET, T 515 LP, and 535/30 ET; and FM4-64, 562/40 HC, HC BS 593, and 641/75 HC. Electronic data were processed using ImageJ (W. Rasband, National Institutes of Health, USA), which also allows the calibration of fluorescence intensity, and of pixel size to determine cell length.

Time-lapse movies were acquired with an interval of 10 s at low laser intensity. For this purpose, cells were mounted on a thicker agarose pad enclosed in a small chamber to avoid desiccation. For microscopy of spores, the same setup was used but images were acquired every 90 s for a minimum period of 2 h. Prior to analysis, maximal-intensity z-projections of three-dimensional (3D) image stacks were aligned using the translation function from the ImageJ StackReg plugin (56) and cropped with ImageJ geometric operations. The movies were processed with Fiji (57).

### Generation of tracks and analysis.

For each condition, around 50 pairs of separating origins were tracked and the tracks were analyzed. Tracks were extracted with TrackMate, a plugin for Fiji (57) that provides a way to semiautomatically segment spots or roughly spherical objects from a 2D or 3D image and track them over time. It follows the classical scheme, where the segmentation step and the particle-linking steps are separated. To follow the origin region separation, pairs of origins in the acquired movies were followed from the time they were overlapping, which was determined by a fluorescence level twice as high as that for single origins, or when they were in close proximity (less than 0.2 μm of separation), until the end of the movie. Movies had a length ranging from 40 min to 1 h. The obtained tracks were then analyzed using MATLAB software.

### Simulation of origin segregation.

The bacterial chromosome has a length of roughly 1 mm and has to fit in a nucleoid with a length of few micrometers. Thus, the chromosome has to be highly compacted (10^3^- to 10^4^-fold). This compaction is achieved by the combined action of specific proteins and the physical effect of macromolecular crowding, which leads to nucleoid compaction as a result of entropic forces between the chromosome and various crowding molecules in the cell (26–29). Additionally, the chromosome is organized into topologically isolated domains, with fluid transitions between domain positions (58–60). The chromosome was found to be divided into 12 to 80 large domains, having a size in the range of 25 kb to 100 kb, or into many more domains of around 10 kb on average. For our simulations we use a model for compacted DNA in which it is assumed that proteins compact DNA, giving it the shape of a chain of beads of diameter $d_{b}$ (21, 22, 61). Within this bead-spring model the chromosome is represented by a chain of 80 beads. Thus, the chromosome with a length of 4 Mb is divided into 80 domains of 50 kb, which is in the range of the above-mentioned domain sizes. For this rather small number of particles molecular dynamics (MD) simulations can be performed. Another limiting point for the simulations is the fact that the time scale of entropic segregation of confined ring polymers is dominated by the so-called “induction phase” (62). This is the time needed to break the initial symmetry. Within this time, a configuration is formed in which each polymer overlaps at one of the sides and a designated direction of segregation is formed. Only after such configurations have been formed do the polymers start separating. It has been shown that the induction phase scales exponentially with chain length (62), which results in an enormous computational cost for simulations with large numbers of beads per chromosome. The MD simulations (where self-avoidance is implemented via repulsive interactions) can be used to study the segregation dynamics. Using MD simulations has the advantage that in principle a real-time scale can be calculated using the energy, length, and mass scales of the analyzed system. As it became apparent, in our simulations replication took much longer than segregation, allowing us to effectively rescale time to cover the complete replication period of 60 min for the chromosomes (see Text S1 for details). Furthermore, space does not have to be discretized and the cellular volume is implemented as a cylindrical compartment of radius $r=5.64\;d_{b}$ (=0.42 μm) and length $l=43\;d_{b}$ (=3.23 μm), which doubles its volume during replication. During this process, the length increases to 5.10 μm and the radius increases to 0.45 μm.

These values are within the range found in the literature (63).

The entropic blob-blob interactions we approximate by a repulsive Debye-Hückel potential:

(1) $$U_{b}\left(r\right)=\epsilon \frac{d_{b}}{r}\text{exp}\left(-\frac{r}{d_{b}}\right),$$

where ϵ parameterizes the strength of the interaction.

The diameter of a bead, *d_b_*, is the basic length scale within the numerical simulations. In combination with the number of beads used to represent the chromosome (*N_b_*) and the resulting mass of a single bead (*m_b_*), one can calculate the time scale of the MD simulations as shown in Text S1.

For the interactions between beads and cell membrane, we use a Weeks-Chandler-Andersen (WCA) potential corresponding to the repulsive part of the Lennard-Jones (LJ) potential (64).

(2) $$V_{\mathrm{WCA}}\left(r\right)=\left\{ \begin{array}{c} 4\varepsilon ({\left(\frac{\sigma }{r-r_{\mathrm{off}}}\right)}^{12}-{\left(\frac{\sigma }{r-r_{\mathrm{off}}}\right)}^{6}+c_{\mathrm{shift}}),\\ 0, \end{array}\right.\begin{array}{c} \text{if}\;r_{\min}+r_{\mathrm{off}}<r<r_{\mathrm{cut}}+r_{\mathrm{off}}\\ \text{otherwise} \end{array}$$

In this study, we chose the parameters as $\sigma =d_{b}=1$, which is the diameter of the beads, the cutoff value $r_{\mathrm{cut}}=1.2246=2^{1/6}\sigma$, which turns the LJ potential into a WCA potential, $c_{\mathrm{shift}}=0.25\varepsilon$ to ensure that the potential is continuous at the cutoff radius and $r_{\mathrm{off}}=0$. With this interaction turned on, the beads were not able to penetrate the wall of the cell.

Furthermore, the beads are connected by elastic springs with a harmonic potential with spring constant *k* that is chosen in such a way that in equilibrium (at extension $r_{0}=d_{b}$) the elastic energy compensates the repulsive Debye-Hückel potential. Larger values for ϵ lead to a stiffer polymer chain with larger *k*. In the simulations we set $\epsilon =1\;k_{B}T$, implying $k=0.74\frac{k_{B}T}{d_{b}^{3}}$. This is a value typically used in MD simulations with Coulomb interactions. Similar values are used in other studies, such as that of Minina and Arnold (62; see also reference 65). We also varied ϵ and found that our results are robust with respect to small variations in ϵ (see Text S1).

The MD simulations were carried out with the simulation package ESPResSo (66). Equations of motion were integrated with the Velocity-Verlet MD integrator with a fixed time step of $t_{\mathrm{step}}=0.01\tau$. A Langevin thermostat was used to keep the system at constant temperature. The friction coefficient was set to $\gamma =\frac{5\epsilon m_{b}d_{b}}{\tau }$. This choice ensured that the motion was overdamped. Variations of γ had only a weak influence on the results.

The MD simulation consists of three parts.

1. To generate a representative ensemble of starting configurations, the cellular volume is discretized and represented by a cubic lattice with lattice constant $d_{b}$. On this lattice a circular self-avoiding random walk is generated, where each of the *N* steps represents a DNA-bead. This walk is then extended to the desired length whereby moves that lead to multioccupation of bonds are discarded to uphold self-avoidance. This allows us to define the *ori*- and *ter*-position in the beginning of the simulation. First, a short and simple self-avoiding walk (SAW) is constructed with fixed *ori* and *ter*. When the SAW has the desired length, the walk is projected onto the confining cylindrical compartment of radius $r=5.64d_{b}$ and length $l=43d_{b}$ used in the MD simulations. The resulting chain of beads is then equilibrated for $10^{3}$ time steps (see Text S1).
2. The DNA strand is duplicated bead by bead with an overall replication time of 3,600 s by two replication polymerases that move along the chromosome in opposing directions starting from *ori*. Therefore, the replication time is divided into intervals in which two new beads are replicated by the two duplication forks. Within these time intervals, the new beads increase their size to the values of the old beads. It is assumed that newly built DNA is compacted right after synthesis. The length of the replication intervals is systematically varied as explained in Text S1. While the beads are grown the complete system is integrated and the chromosomes begin to separate already during duplication due to the thermal fluctuations and the resulting entropic repulsion between the full chromosome and the partially replicated chromosome. For the position of replication forks, we implemented two different models: (i) the track model (13), where the polymerases move along the chromosome, and (ii) the factory model (see reference 15 and Text S1 for more information), where replication occurs at a fixed cellular position.
3. After duplication, the MD simulations run until the chains are completely segregated. The degree of separation is quantified by the longitudinal overlap of the chromosomes within the cell divided by the longitudinal elongation of the shorter chromosome in the cell.

An essential challenge in processing MD simulations for large chains of beads is the computational cost of the simulations. Thus, in order to simulate a process with a duration of almost 1 h, the initially shorter simulation times had to be rescaled to realistic simulation times. This was possible because of the dominance of the replication period over the segregation period with respect to the time scale. The exact procedure and motivation for this rescaling are explained in Text S1.
