# Supplementary figures and images for: Chromosome Segregation in Bacillus subtilis Follows an Overall Pattern of Linear Movement and Is Highly Robust against Cell Cycle Perturbations
Source: mSphere. 2020 Jun 17;5(3):e00255-20. doi: 10.1128/mSphere.00255-20 (PMC7300352; doi:10.1128/mSphere.00255-20)

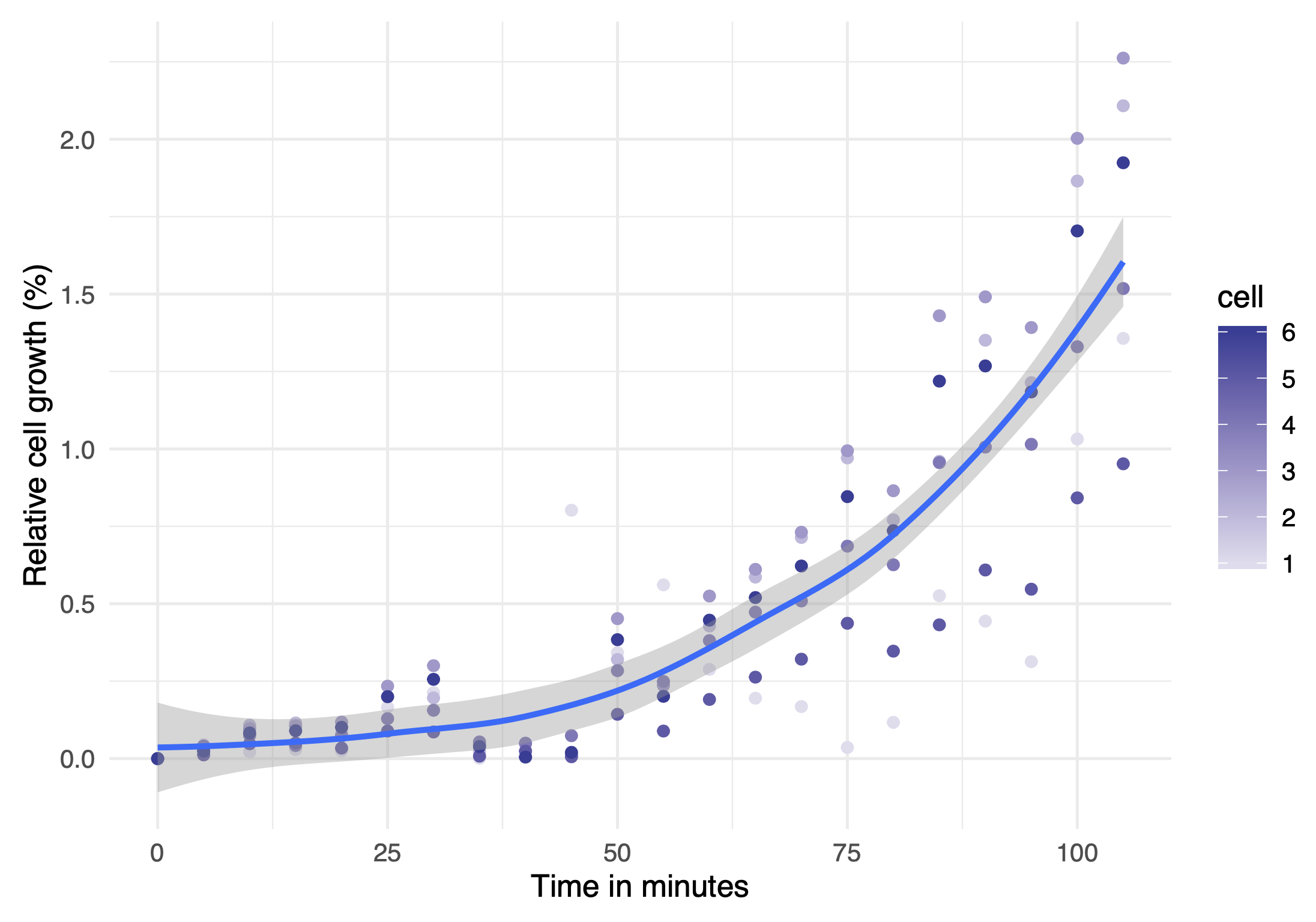

Supplement: FIG S1 [file mSphere.00255-20-sf001.tif]

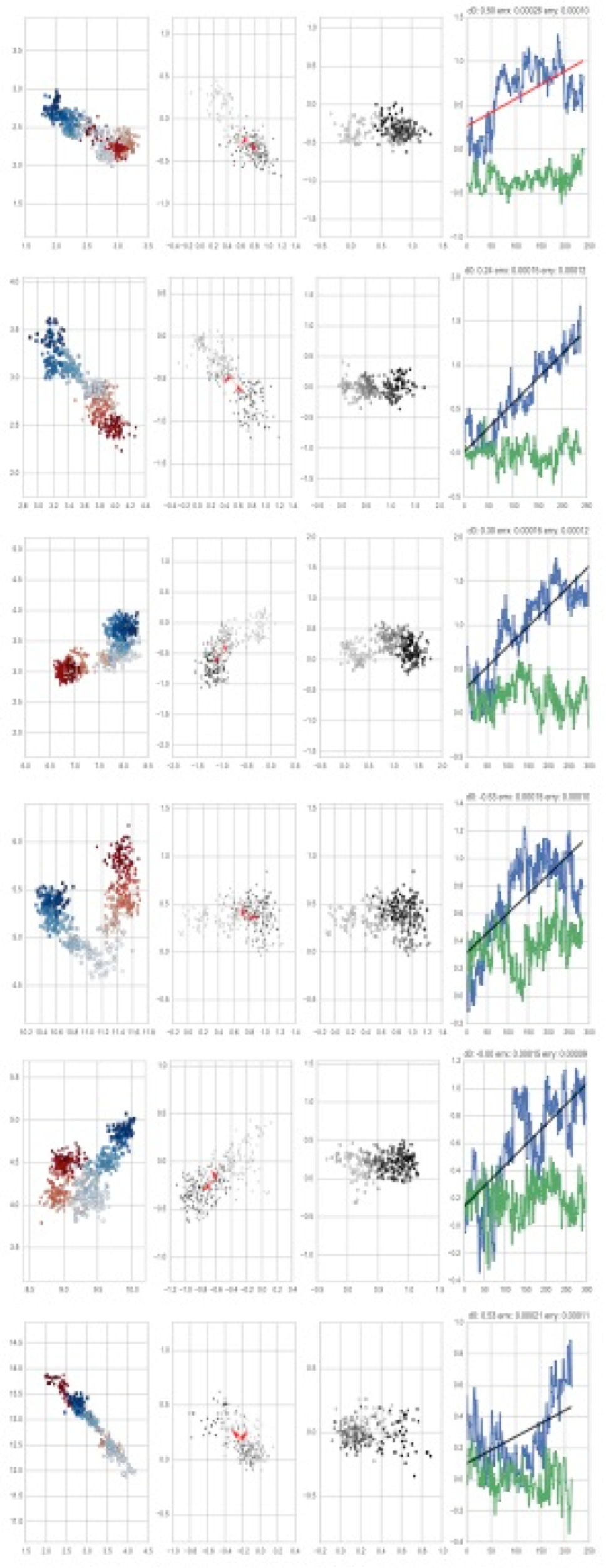

Supplement: FIG S2 [file mSphere.00255-20-sf002.tif]

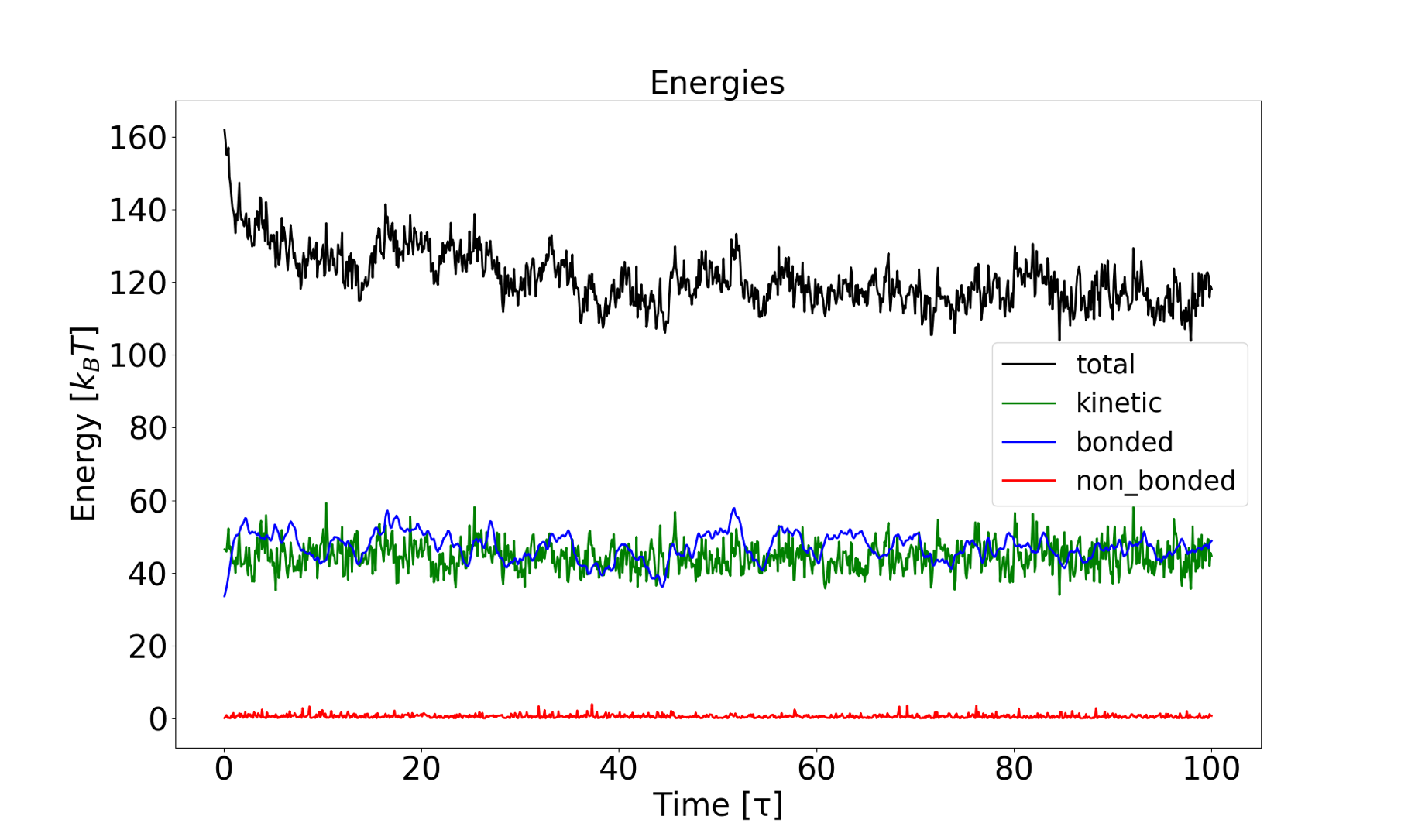

Supplement: FIG S3 [file mSphere.00255-20-sf003.tif]

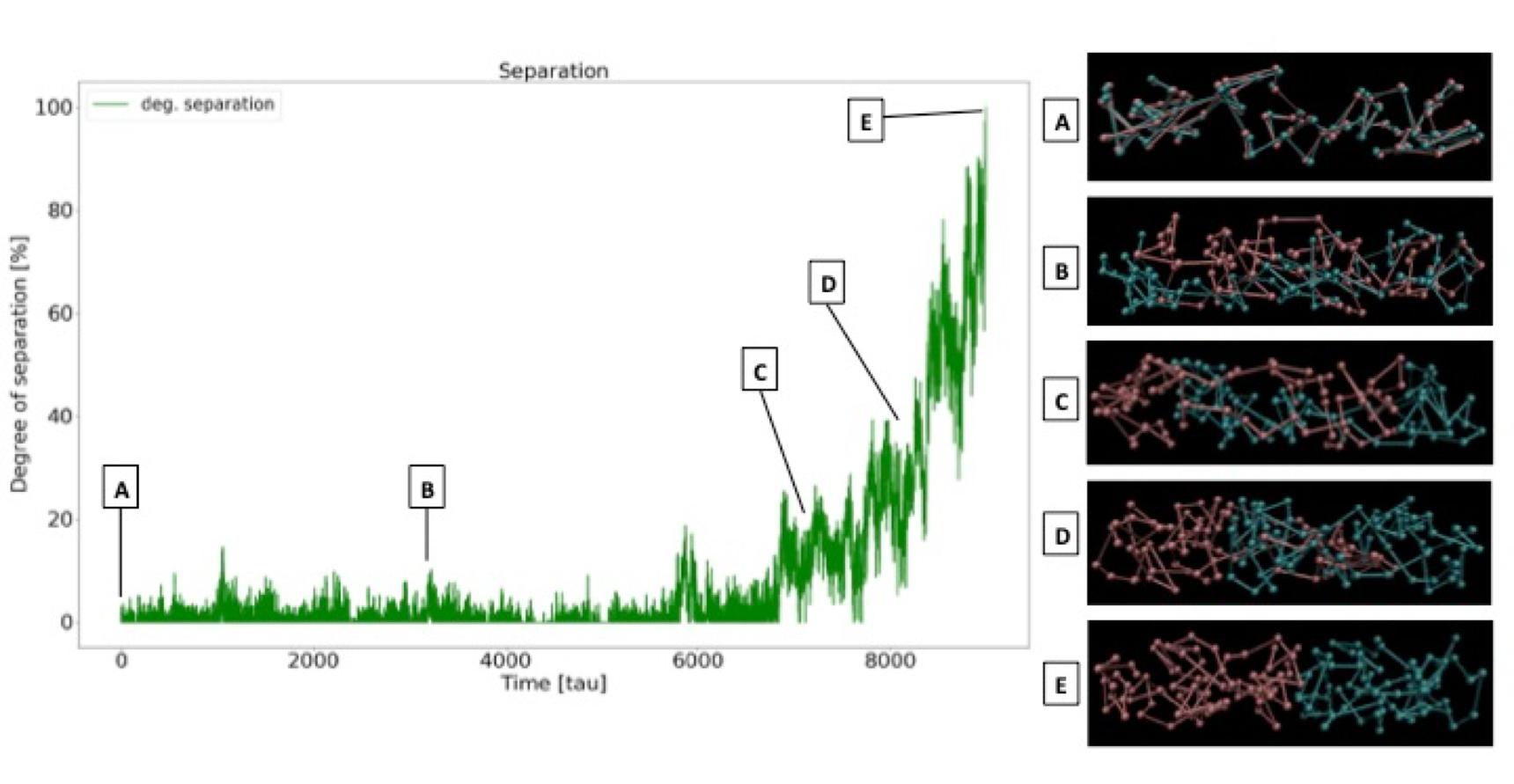

Supplement: FIG S4 [file mSphere.00255-20-sf004.tif]

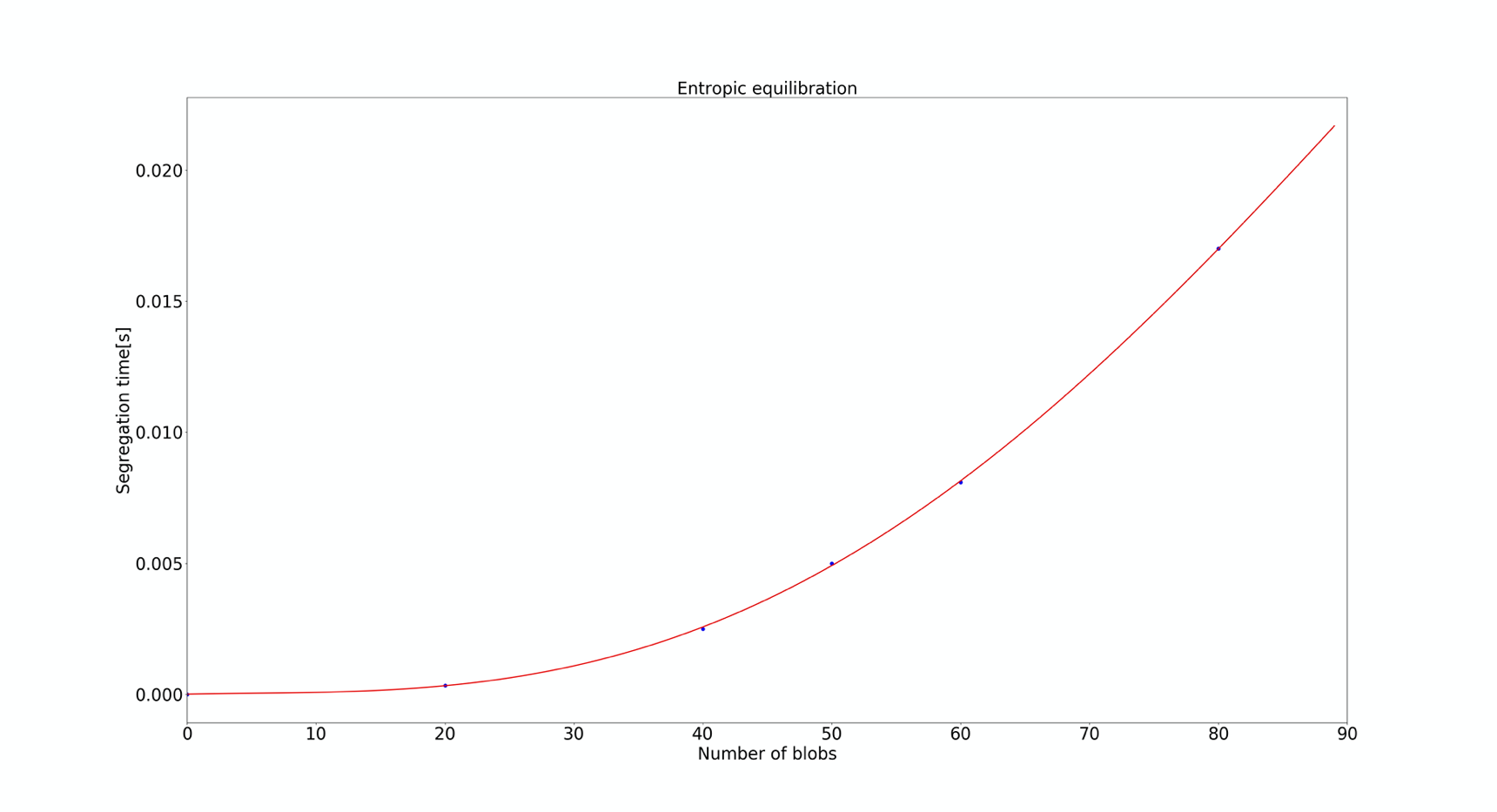

Supplement: FIG S5 [file mSphere.00255-20-sf005.tif]

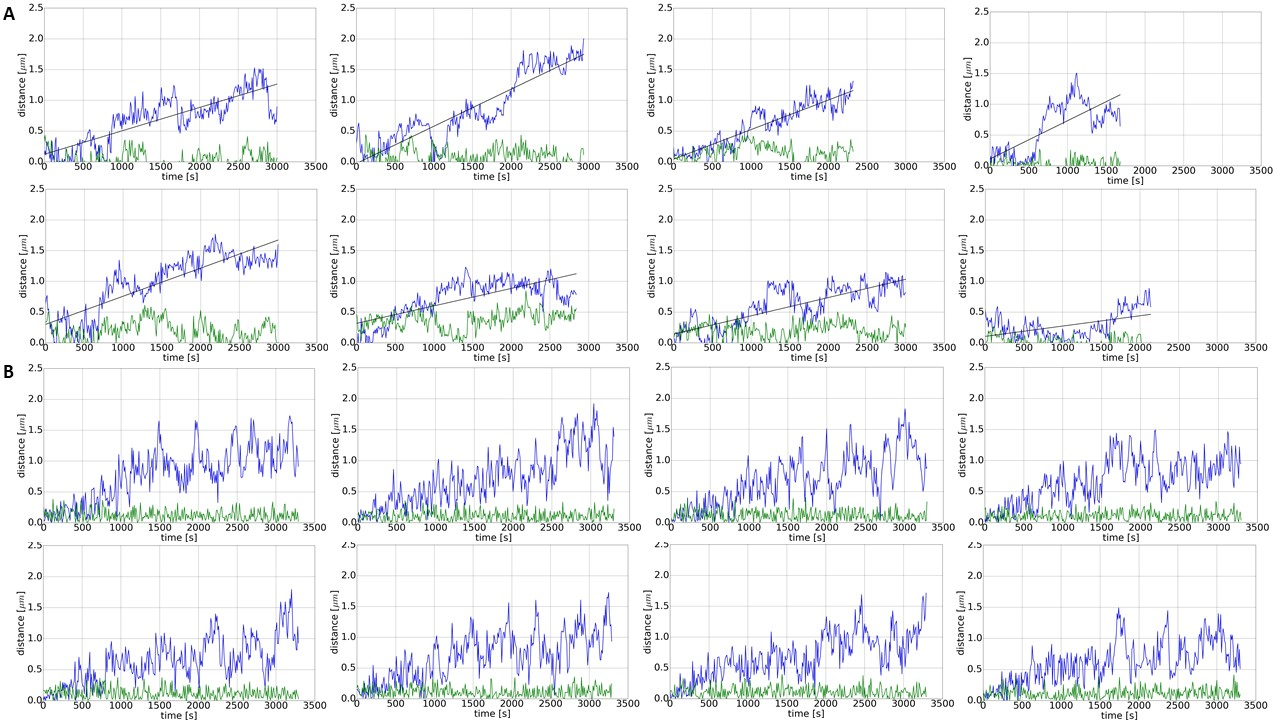

Supplement: FIG S6 [file mSphere.00255-20-sf006.tif]

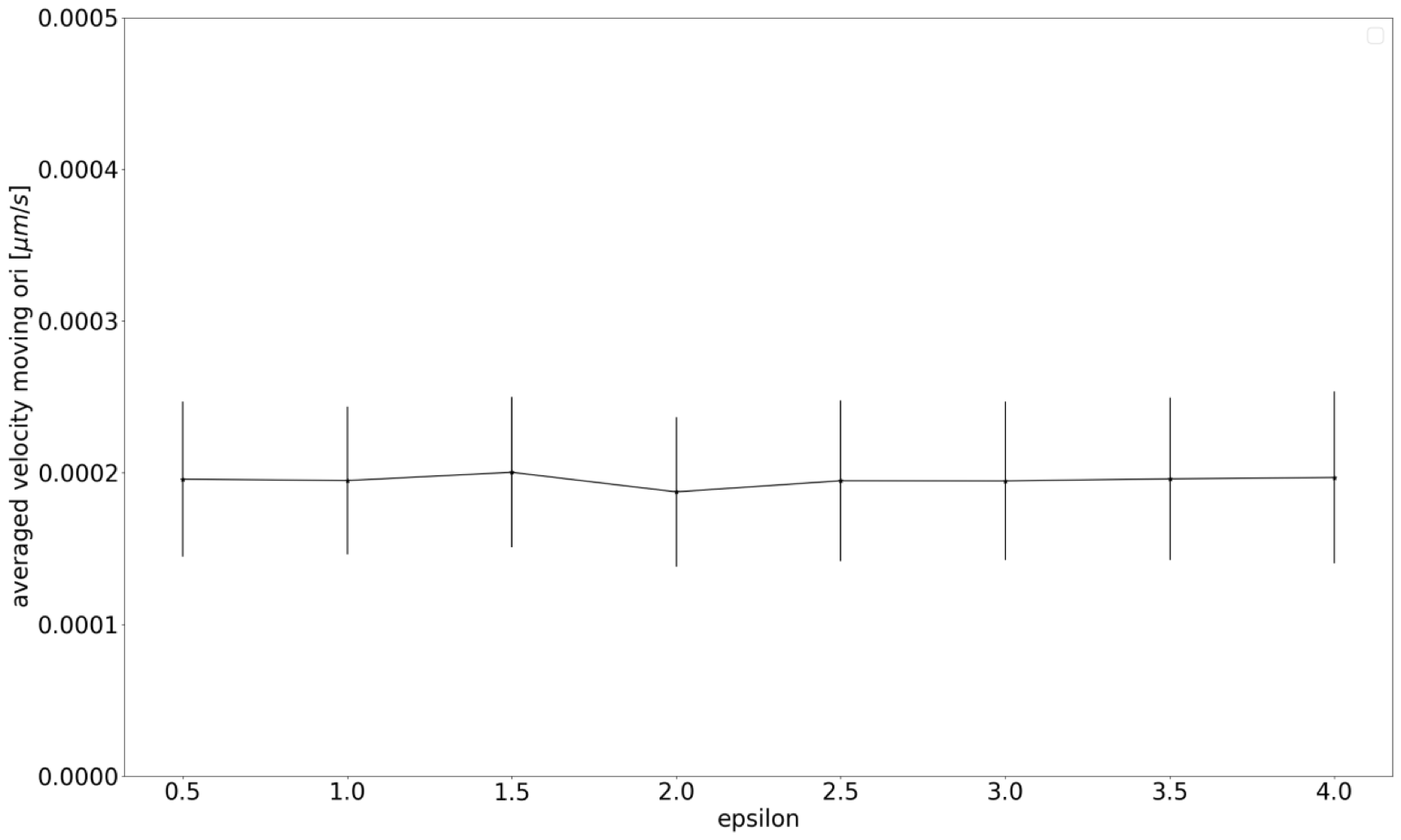

Supplement: FIG S7 [file mSphere.00255-20-sf007.tif]

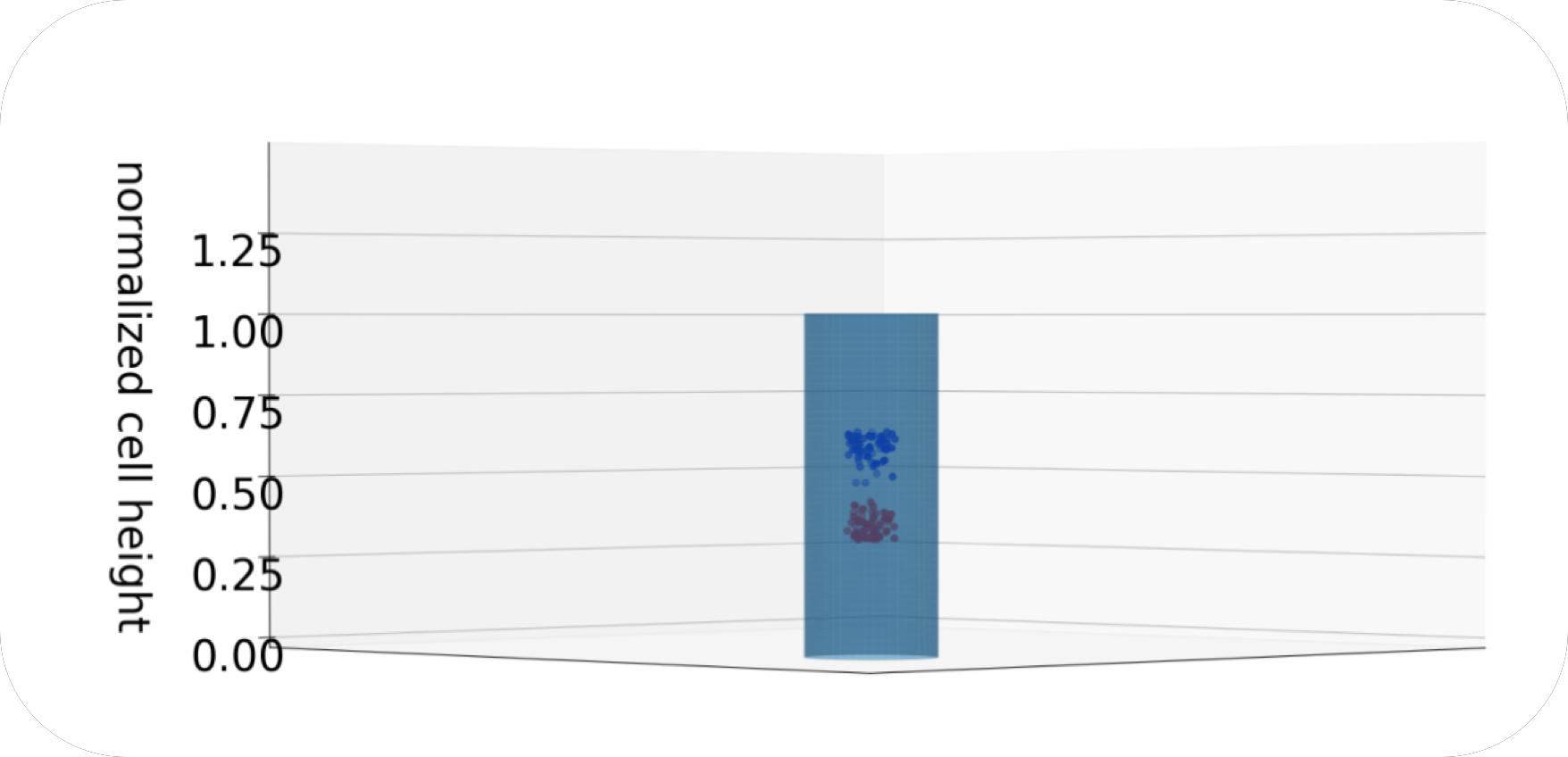

Supplement: FIG S8 [file mSphere.00255-20-sf008.tif]
