# Supplementary material for: Chromosome Segregation in Bacillus subtilis Follows an Overall Pattern of Linear Movement and Is Highly Robust against Cell Cycle Perturbations
Source: mSphere. 2020 Jun 17;5(3):e00255-20. doi: 10.1128/mSphere.00255-20 (PMC7300352; doi:10.1128/mSphere.00255-20)
Supplement: TEXT S1 [file mSphere.00255-20-s0001.docx]

**Supplementary Methods**

**Calculation of time scale within MD simulations**

The software package ESPResSo used for the MD simulations does not predefine any units in contrast to many other MD programs. Instead, the user has to define the length-, time- and energy scales.

The basic length scale of our simulations is the blob diameter *d_B_* = 75nm. Therefore, all lengths are measured in multiples of *d_B_*:

$$\left[ length \right]= 75 nm$$

The energy scale is given by the binding energy of the particles, i.e. the value of the Debye-Hückel potential for the bond length *d_B_*, *V_DH_*(*d_B_*) = 0.368k_B_T. At this length the repulsive Debye-Hückel potential and the attracting harmonic potential of the springs exactly compensate each other. All energies are measured in multiples of this energy

$$\left[ energy \right]= 0.368 k_{B}T$$

The mass of the system is given by the mass of the particles in the system, which can be calculated by the content of base pairs (bp). One bp of DNA has a weight of 650 Da. The genome of *B. subtilis* has about 4 Mbp of DNA. Thus, the mass of the whole genome is roughly 4.316*10^-18^ kg. In our simulations the DNA is packaged within compact domains called beads. They are the particles within our system and the weight of one bead depends on the degree of compactification of the DNA, i.e. the answer to the question into how many blobs the 4 Mbp of DNA are packaged.

$m_{B}= \frac{4 Mbp*650 \frac{Da}{bp}}{Blobs per chromosome}$

Finally, we can calculate the resulting time scale with

$$\left[ time \right]=\left[ length \right]\sqrt{\frac{\left[ mass \right]}{\left[ energy \right]}}=75 nm \sqrt{\frac{m_{B}}{0.368 k_{B}T}}$$

Here, *m_B_* denotes the mass of one of bead. The thermal energy is given by the Boltzmann constant k_B_ and the temperature T.

If we plug in some typical values into the equation above, for example 80 blobs per chromosome and setting the temperature to room temperature of 293 K, we obtain

$\tau=75*{10}^{-9}m* \sqrt{\frac{5.4*{10}^{-20}kg}{0.368*1.38*{10}^{-23}\frac{J}{K}*293K}}=4.51*{10}^{-7}s$ *

as the basic time step of our simulation.

Furthermore, we notice that in order to simulate a replication period of 60 min we would have to calculate about ${8*10}^{9}$ simulation steps. This exceeds the computational power by far and thus demands a possibility of accelerating our simulations.

**Entropic Equilibration of confined polymers**

In order to find a possibility to accelerate our simulations we compared the time scales of replication and segregation of chromosomes within our model. For this purpose, we performed simulations of initially mixed up chromosomes and measured the time for entropic segregation. An example run of such a simulation with snapshots of the chromosomes is shown in figure S4:

As can be seen in figure S4 the chromosomes typically need a so called “induction phase” (1) before segregation sets in. The induction phase is due to the fact that before segregation can start, the initial system symmetry has to be broken by spontaneous fluctuations (1). The length of the induction phase scales exponentially with the length of the polymers and thus can dominate the overall segregation time for long polymers (1). After the induction phase, segregation proceeds rather rapidly until the chromosomes are fully separated.

We performed such simulations for varied numbers of blobs per chromosome as shown in figure S5. As can be seen here, the time for complete segregation of two chromosomes is of order of some ms and thus very short. Therefore, we can conclude that within our model the time scale is dominated by the replication time of around 60 min.

Furthermore, we can apply a polynomial fit to the results of figure S5 as shown by the red line. Thereby, we can interpolate the time for entropic segregation of two beads, $t_{Equi,Model}\approx195 \mu s$, giving a time scale for the separation of a newly replicated part of the chromosome.

The reason for this is that in the simulations replication consists of a succession of steps in which two blobs from the mother chromosome are duplicated and the new blobs of the daughter chromosome begin to separate from the mother chromosome. In this process, they are hindered by the replication forks attaching them to the old chromosome. Consequently, we do not expect anything new to happen after integrating the model more than $t_{Equi,Model}\approx195 \mu s$ after a duplication step because entropic equilibration of the new blobs should be accomplished already. Hence, we can accelerate our simulations at this point by jumping to the next duplication step.

In order to rescale our simulation time after such an acceleration, we have to compare the entropic equilibration time for two blobs in our model with the one we would expect in an experiment, if replication was divided into the same amount of duplication steps:

$$t_{Equi, Exp}=\frac{t_{rep}}{( \frac{N_{B}}{2})}$$

Here, N_B_ represents the number of blobs of one complete chromosome and *t_rep_* the time for replication of the complete chromosome. Because two polymerases move along the chromosome, the replication time is divided into $\frac{N_{B}}{2}$ duplication steps.

Following these considerations, we get rescaling factors depending on the number of blobs of a chromosome

$$f_{rescale}(N_{B})=\frac{t_{Equi,Exp}}{t_{Equi, Model}}$$

**Implementation of different replication models**

Within the numerical simulations we implemented two different models for replication (see Fig. 3 for snapshots). Within the so-called “track model” replication is not fixed to a specific point in the cell. Instead, the replication polymerases move along the chromosome like a train on a track (2). Therefore, in the numerical simulations replication starts at *ori* and the first two beads are replicated in the first duplication step (see first snapshot in right panel of Fig. 3). In the further course of replication within each duplication step the next two adjacent beads are replicated and the complete system is integrated between duplication steps, thereby changing the positions of the beads (see following snapshots of right panel in Fig. 3). Thereby, the movement of the two polymerases in opposing directions along the chromosome is modelled. At the end of replication the old chromosome is completely duplicated (see last snapshot of right panel in Fig. 3).

In contrast, within the “factory model” replication is fixed to a cellular region at midcell where the replication polymerases are positioned like a factory through which the DNA is pulled (3, 4). While for example in *E. coli* it is not clear, whether a replication factory exists or not, for *B. subtilis* it was shown that such a factory is localized near midcell, thus supporting the idea of the factory model (2, 5).

In our simulations we modelled the replication factory as an additional spatially fixed bead at midcell (grey bead in snapshots of left panel in Fig. 3), which is connected via springs to the next beads of the old chromosome to be duplicated. Thereby, it is assured that replication happens at midcell and that the beads next to be duplicated are pulled in the proximity of the replication factory. Thus, within the factory model the old chromosome is duplicated at midcell and the daughter strands start segregating from there (see snapshots on left panel of Fig. 3) until the old chromosome is completely pulled through the replication factory and replicated (last snapshot of left panel of Fig. 3).

**Variation of replication intervals**

As explained above the total replication time of 3600 s is subdivided into replication intervals in which two beads are duplicated within our model. Within these replication intervals the newly duplicated beads grow until they reach the same mass and charge as the beads from the old chromosome. Because the complete replication period has a duration of 3600 s and within each period two new beads are duplicated, the single replication intervals must have an average duration of

$$\Delta t_{rep}= \frac{3600 s}{blobs per chromosome}*2$$

By letting the mass and charge of newly duplicated beads grow within a replication period we aimed to model the experimental situation as good as possible. However, the sudden emergence of new beads (possibly rather near to the old beads) may result in unphysical repulsion forces between the beads for small durations in which the beads must equilibrate. To prevent a systematic error of our simulations due to this fact we modulated the length of the replication intervals by varying their duration with a gaussian distribution. Thereby, the possible equilibration errors are canceled out and we still end up with a correct average duration of the replication period of 60 min.

**Variation of** $\boldsymbol{\epsilon}$ **within simulations**

To analyse the effect of changing the value of $\boldsymbol{\epsilon}$ within our simulations we performed simulations with $\epsilon$ ranging from 0.5 - 4 (see Fig. S7).

We checked the average velocity of the faster moving *ori* along the longitudinal axis of the cell to observe any changes in its movement as a result of the changed parameter. Within the tested range no significant changes arise due to the diversification of $\epsilon$. Thus, we conclude that our model is robust concerning small variations of $\epsilon$.

**Spatial organization of origins**

We also analysed the results of the simulations for the final positions of the two origins after replication (Fig. S8). There exist two distinct patterns of spatial organization concerning the origins within bacteria, the so called “*ori-te*r” pattern where newly replicated origins reside at different cell poles and the “left-*ori*-right” pattern where the origins are found at the 1/4 and 3/4 positions along the longitudinal axis of the cell (6). For *B. subtilis* it was shown that the chromosome alternates between the two patterns (6). We found that the average positions (averaged over 80 runs) of the origins in our model were 0.38 and 0.61. Thus, the oris are slightly more centralized in our model than in experimental findings of the “left-*ori*-right” pattern. Therefore, we conclude that the exact positioning of the origins may require an additional mechanism. This is in agreement with recent findings of Hofmann et al. (2019) who found in simulations of *E. coli* that entropic repulsion alone is not sufficient for both timely and accurate segregation and positioning of *oris* but that also preferential loading (by MukBEF) was needed to place the *oris* at the expected quarter cell positions (7).

**References**

1. Minina E, Arnold A. 2014. Induction of entropic segregation: the first step is the hardest. Soft Matter 10:5836-5841.

2. Lemon KP, Grossman AD. 1998. Localization of bacterial DNA polymerase: evidence for a factory model of replication. Science 282:1516-1519.

3. Badrinarayanan A, Le TB, Laub MT. 2015. Bacterial chromosome organization and segregation. Annual Review of Cell and Developmental Biology 31:171-199.

4. Lemon KP, Grossman AD. 2001. The extrusion-capture model for chromosome partitioning in bacteria. Genes Dev 15:2031-2041.

5. Lemon KP, Grossman AD. 2000. Movement of Replicating DNA through a Stationary Replisome. Mol Cell 6:1321-1330.

6. Wang X, Montero Llopis P, Rudner DZ. 2014. *Bacillus subtilis* chromosome organization oscillates between two distinct patterns. Proc Natl Acad Sci U S A 111:12877-12882.

7. Hofmann A, Makela J, Sherratt DJ, Heermann D, Murray SM. 2019. Self-organised segregation of bacterial chromosomal origins. Elife 8.
